# High-resolution ultrasound as an aid in the diagnosis and treatment of post-brachioplasty injury to the medial brachial and the medial antebrachial nerves – Two case reports^☆^

**DOI:** 10.1016/j.ijscr.2020.06.043

**Published:** 2020-06-14

**Authors:** Mohammad M. Al-Qattan, Ahmed K. Thallaj

**Affiliations:** a Department of Surgery, King Saud University, Riyadh, Saudi Arabia; b Department of Anesthesia, King Saud University, Riyadh, Saudi Arabia

**Keywords:** High-resolution ultrasound, Brachioplasty, Injury, Medial brachial nerve, Medial antebrachial nerve

## Abstract

**Introduction:**

Injury to the medial brachial and the medial antebrachial nerves may occur after brachioplasty and may lead to severe pain. We report on two cases to demonstrate that high-resolution ultrasound may be used as an aid in the diagnosis and treatment of post-brachioplasty injury to these small cutaneous nerves.

**Case reports:**

We report on two cases. The first case was a 29-year old female with severe arm pain secondary to a neuroma of the right medial brachial nerve. The diagnosis of a neuroma was done using high-resolution ultrasound and proximal nerve block led to immediate complete relief of pain. Recurrent but milder pain occurred and this was treated with multiple nerve blocks. The second case was a 34-year-old female with severe arm/forearm pain secondary to entrapment of the left medial antebrachial cutaneous nerve by a suture in the middle arm. The diagnosis was reached using high-resolution ultrasound and proximal nerve block led to immediate complete relieve of pain. Recurrent but milder pain occurred and this was treated by surgical nerve decompression.

**Discussion:**

Previous literature confirms that high-resolution ultrasound can accurately identify small sensory nerves of the arm and forearm.

**Conclusion:**

We demonstrate that high resolution ultrasound may be used as an aid in the diagnosis and treatment of post-brachioplasty injury to the medial brachial and the medial antebrachial nerves. Proximal nerve block is extremely effective to relief the severe pain in these patients. However, recurrence of pain is expected and further management may be done by a trial of repeated injections or by surgical exploration.

## Highlights

• High-resolution ultrasound can accurately identify small sensory nerves of the arm and forearm.
• Nerve injury may occur following brachioplasty.
• One patient had neuroma of the medial brachial nerve with severe pain.
• Another patient had entrapment of the medial antebrachial nerve with severe pain.
• High-Resolution ultrasound was used an aid in the diagnosis and blocking of the nerves.

## 1 Introduction

With the growth of bariatric surgery, there is more demand for body-contouring surgery including brachioplasty. Brachioplasty is done to remove the excess, bat-wing-like skin of the arm, which may be of variable severity [1]. Potential complications of the brachioplasty procedure include under-resection, over-resection, asymmetry, seroma, hematoma, infection, dehiscence, skin necrosis, distal edema, dog ears, scar contracture, hypertrophic scars and nerve injury [2]. Nerve injury is relatively rare, occurring in about 1.5% of all previously reported brachioplasty procedures [2]. The most common nerve injury is the medial antebrachial nerve (also known as the medial cutaneous nerve of the forearm) followed by compression of the ulnar nerve [2,3]. Injury to the medial brachial cutaneous nerve (also known the medial cutaneous nerve of the arm) is extremely rare [4].

We present a case of a painful neuroma of the medial brachial cutaneous nerve post brachioplasty and another case of entrapment of the medial antebrachial nerve in a surgical suture. In both cases, the diagnosis and treatment were aided with the use of high-resolution ultrasound.

The previous literature only showed the feasibility of high-resolution ultrasound in the identification of small cutaneous nerves. Thallaj et al. [5] described in human volunteers the technique for ultrasound identification of the medial antebrachial cutaneous nerve and the technique for ultrasound-guided blockade of this sensory nerve. Moritz et al. [6] confirmed the ability of high-resolution ultrasound to visualize and infiltrate small subcutaneous nerves of the forearm in anatomic specimens. In the current paper we show that the ultrasound is also helpful in the diagnosis and treatment of injuries of these small cutaneous nerves. The work has been reported in line with the SCARE Criteria [7].

## 2 Case reports

### 2.1 Case #1: injury to the medial brachial cutaneous nerve

A 29-year old, otherwise healthy, female presented with severe neuropathic pain and hypersensitivity in the right arm as a complication of brachioplasty. She has been treated with various analgesics with little benefit. She presented to us 17 months after the brachioplasty procedure. Examination showed a positive Tinel sign at the junction of the middle and upper thirds of the arm along the incision line. The sensory dermatome of the medial antebrachial nerve was normal (by comparing it to the contralateral asymptomatic left side). Functional assessment of the main nerves of the limb (medial, ulnar, and radial nerves) revealed no abnormalities. Examination of the hand did not show any signs of complex regional pain syndrome. The patient scored the pain as10 out of 10 without the analgesics and 8 out of 10 with the use of analgesics. The clinical impression was neuroma of the medial brachial cutaneous nerve.

**Fig. 1:**
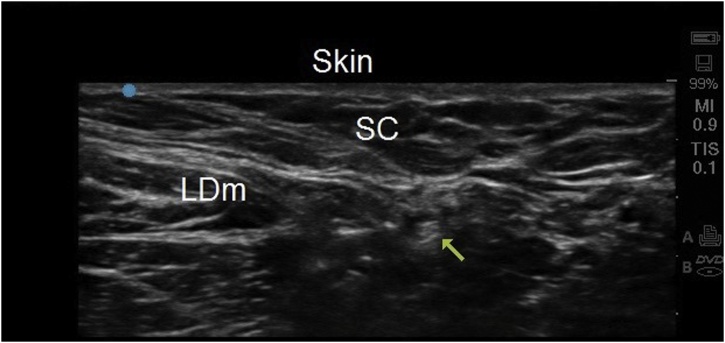
Cross-sectional view at the level of axilla near the scar. The arrow points at the medial brachial cutaneous nerve where it was injected proximally. SC: Subcutaneous tissue; LDm; Latissimus Dorsi muscle.

**Fig. 2:**
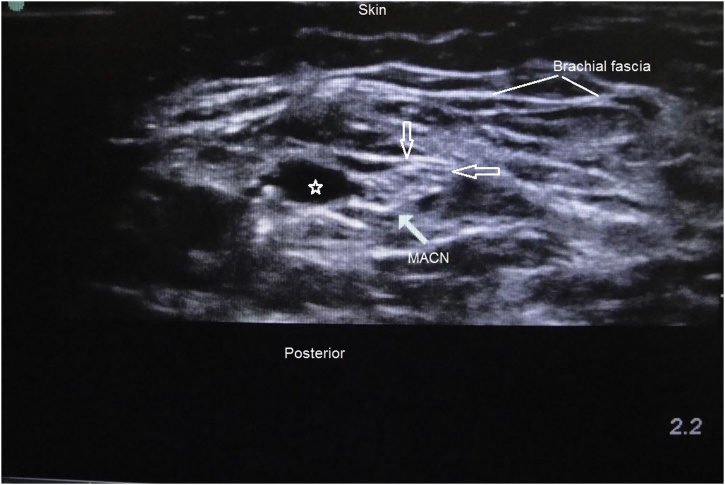
Cross-sectional sonographic view of the arm. Arrows point to the medial antebrachial cutaneous nerve (MACN) proximally. The star is at the nerve just distal to the entrapment site; showing the abnormal hyo-echoic surrounding indicating perineural edema.

The patient was placed in semi-lateral position with the arm abducted and elevated above the head level. An Ultrasound scan was performed using a 6–15 MHz, 50-mm linear probe (Sonosite M-Turbo; Sonosite Inc., Bothell, WA, USA). The probe was positioned proximal to the base of the axillary fossa in a transverse plane, between the pectoralis major and latissimus dorsi muscles. The medial brachial cutaneous nerve was identified and followed distally. At the area of maximum pain, disruption of nerve continuity and a neuroma were identified. The nerve was located proximally (Fig. 1) and 2 mL Bupivacaine 0.25% (Marcaine, Astra Zeneca Pharmaceuticals) and 1 mL of 40 mg/mL of Methylprednisolone acetate (Depo-Medrol, Pfizer, INC. New York, NY) was injected around the nerve.

Immediately after the nerve block, there was complete relief of symptoms (pain score zero out of ten). At two weeks after the injection, her pain score was 2 out of 10 (with no analgesics). She then travelled to her home town and was contacted by phone afterwards. About 4 weeks after the injection, slight worsening of the pain (a pain score of 4 out of 10 with no analgesics) was noted. The patient underwent repeated injections at her home town.

### 2.2 Case #2: entrapment of the medial antebrachial nerve

A 34-year old, otherwise healthy, female presented to us 18 days post bilateral brachioplasty done outside of the country. Immediately after the surgery, she noted numbness along the distribution of the left medial antebrachial nerve. There was also severe pain in the arm along the incision site that radiated to the medial aspect of the forearm (pain score 9 out of 10). Pain increased (a score of 10 out of 10) with simultaneous shoulder abduction and elbow extension. She has been treated with various analgesics with little benefit. Examination showed loss of sensation along the distribution of the medial antebrachial nerve. Demonstration of the Tinel sign was difficult because of the pain. Functional assessment of the main nerves of the limb (medial, ulnar and radial nerves) revealed no abnormalities. Examination of the hand did not show any signs of complex regional pain syndrome.

Using the same technique and the same Ultrasound machine used in the first case, the medial antebrachial nerve was identified along the entire arm. The nerve was found to be distorted and edematous about 9 cm proximal to the medial epicondyle along the incision indicating its entrapment in a fascial suture (Fig. 2). There was no disruption of nerve continuity. The nerve was blocked above the point of nerve edema using 1 ml of Bupivacaine +1 ml of Depo-medrol.

Immediately after the nerve block, there was complete relief of pain (zero out of 10). Two days later there was mild recurrence of pain (3 out of 10). She was offered surgical exploration to decompress the nerve and remove the fascial suture around the nerve. The patient travelled back to her original surgeon and underwent the procedure with excellent recovery at 6 months (a pain score of 2 out of 10 with no analgesics). The patient also reported partial recovery of sensation along the distribution of the nerve in the forearm.

## 3 Discussion

The medial brachial cutaneous nerve arises from the medial cord of the brachial plexus. It passes medial to the axillary vein in the axilla and upper arm. It then passes medial to the brachial artery in the middle arm to pierce the deep fascia to supply sensation of the medial and posterior aspect of the lower arm reaching the medial epicondyle and the olecranon area. The nerve is also known as the nerve of Wrisberg (after it Heinrich Wrisberg who described the anatomy of the nerve) [8]. Injury of the medial cutaneous nerve post brachioplasty is relatively rare [4].

The medial antebrachial cutaneous nerve also arises from the medial cord of the brachial plexus and lies medial to the axillary artery in the axilla. At the lower axilla the nerve gives a small branch which pierces the fascia to supply the skin over the biceps muscle. The main trunk of the nerve then descents medial to the brachial artery to pierce the fascia in the middle part of the arm. The nerve then terminates in a volar branch (supplying the skin of ulnar side of the forearm) and an ulnar branch (supplying the skin on the posterior-ulnar aspect of the forearm) [9]. Injury to the medial antebrachial nerve is relatively common in surgical practice and may be seen after brachioplasty [2], after brachial plexus blocks [10], after cubital tunnel release [9] and after phlebotomy [11].

High-resolution ultrasound is known to be accurate in the visualization and identification of small cutaneous nerves of the arm and forearm [5,6]. In our paper, we show that high resolution ultrasound is also able to identify the pathology (neuroma versus entrapment in a suture) of the injured nerves following the brachioplasty procedure. Furthermore, proximal nerve block with the aid of high-resolution ultrasound is accurate and is extremely effective to relief the pain. However, recurrence of pain is expected and further management may be done by a trial of repeated injections or by surgical exploration. Once again, if surgical exploration is chosen, the exact site of injury can be located and marked on the skin pre-operatively using the high- resolution ultrasound.

## 4 Conclusions

We demonstrate that high resolution ultrasound may be used as an aid in the diagnosis and treatment of post-brachioplasty injury to the medial brachial and the medial antebrachial nerves. A proximal nerve block is extremely effective to relief the severe pain in these patients. However, recurrence of pain is expected and further management may be done by a trial of repeated injections or by surgical exploration.

## Declaration of Competing Interest

None.

## Sources of funding

None.

## Ethical approval

The study was approved by the research committee, National Hospital, Riyadh, Saudi Arabia.

## Consent

Written informed consent was obtained from both patients for publication of this case report and accompanying images. A copy of the written consent is available for review by Editor-In-Chief of this journal on request.

## Author contribution

Both authors contributed significantly and in agreement with the content of the manuscript. Both authors participated in data collection and in writing of the manuscript. The second author performed the ultrasound.

## Registration of research studies

Not relevant here.

## Guarantor

M M Al-Qattan.

## Provenance and peer review

Not commissioned, externally peer-reviewed.
